# Assessing the acute toxicity of insecticides to the buff-tailed bumblebee (*Bombus terrestris audax)*

**DOI:** 10.1016/j.pestbp.2020.104562

**Published:** 2020-06

**Authors:** Rebecca J. Reid, Bartlomiej J. Troczka, Laura Kor, Emma Randall, Martin S. Williamson, Linda M. Field, Ralf Nauen, Chris Bass, T.G. Emyr Davies

**Affiliations:** a Department of Biointeractions and Crop Protection, Rothamsted Research, Harpenden, UK; b Bayer AG, Crop Science Division, Alfred Nobel-Strasse 50, 40789 Monheim, Germany; c College of Life and Environmental Sciences, Biosciences, University of Exeter, Penryn Campus, Penryn, Cornwall, UK

**Keywords:** Bumblebee, Neonicotinoids, Pyrethroids, Organophosphates, Differential toxicity

## Abstract

The buff-tailed bumblebee, *Bombus terrestris audax* is an important pollinator within both landscape ecosystems and agricultural crops. During their lifetime bumblebees are regularly challenged by various environmental stressors including insecticides. Historically the honey bee (*Apis mellifera spp.) has* been used as an ‘indicator’ species for ‘standard’ ecotoxicological testing, but it has been suggested that it is not always a good proxy for other eusocial or solitary bees. To investigate this, the susceptibility of *B. terrestris* to selected pesticides within the neonicotinoid, pyrethroid and organophosphate classes was examined using acute insecticide bioassays. Acute oral and topical LD_50_ values for *B. terrestris* against these insecticides were broadly consistent with published results for *A. mellifera*. For the neonicotinoids, imidacloprid was highly toxic, but thiacloprid and acetamiprid were practically non-toxic. For pyrethroids, deltamethrin was highly toxic, but tau-fluvalinate only slightly toxic. For the organophosphates, chlorpyrifos was highly toxic, but coumaphos practically non-toxic. Bioassays using insecticides with common synergists enhanced the sensitivity of *B. terrestris* to several insecticides, suggesting detoxification enzymes may provide a level of protection against these compounds.

The sensitivity of *B. terrestris* to compounds within three different insecticide classes is similar to that reported for honey bees, with marked variation in sensitivity to different insecticides within the same insecticide class observed in both species. This finding highlights the need to consider each compound within an insecticide class in isolation rather than extrapolating between different insecticides in the same class or sharing the same mode of action.

## Highlights

• Acute toxicity of popular insecticides to *Bombus terrestris audax* was assessed.
• Compounds from 3 major classes were tested in topical and oral bioassays.
• Bioassays with different synergists were done for each compound.
• Results show stark differential toxicity of insecticides within the same class.

## Graphical abstract

**Figure:**
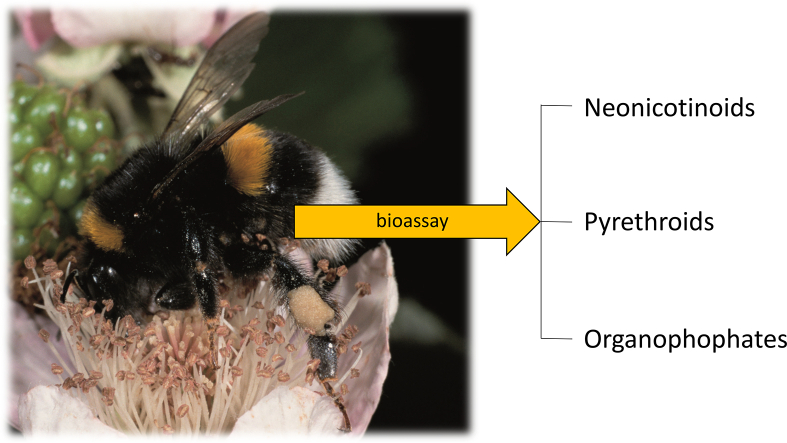

## 1 Introduction

The importance of bees to agriculture and horticulture cannot be overstated. Approximately a third of all crops consumed by humans globally are bee pollinated (Delaplane and Mayer, 2000). It is notoriously difficult to estimate the economic value of the pollination services contributed by bees worldwide, but there is little doubt that it is in the billions of dollars for honey bees alone (Gallai et al., 2009; Lautenbach et al., 2012). Whilst historically the European honey bee, *Apis mellifera*, has been the focus of such estimates, the importance of other wild and managed bee species is increasingly being recognized. Wild insect species are often more efficient pollinators of crops (Garibaldi et al., 2013), and wild bee species provide the same economic contribution to crop pollination as managed honey bees (Kleijn et al., 2015). Across Europe, the top wild bee crop pollinators are *Bombus terrestris / lucorum* complex and *Bombus lapidarius* (Kleijn et al., 2015). *Bombus* species are now also the main alternative to honey bees for commercial pollination in Europe and North America (Goulson, 2010). They are particularly utilized in the production of tomatoes and other greenhouse crops due to their ability to buzz-pollinate (Velthuis and van Doorn, 2006), whilst the longer tongues of some bumblebee species make them better at pollinating flowers with deeper corollas (Plowright and Plowright, 1997). Bumblebees also start foraging earlier in the day (Stanghellini et al., 2002) and continue to forage in cold and wet weather that is unsuitable for honey bees (Corbet et al., 1993), enabling them to provide a more consistent pollination service. Because they are hardier, they are also better able to pollinate wild flowers in remote or fragmented locations, although their impact on wild plant species is less well studied than on agricultural species (Goulson, 2010).

Bee populations worldwide appear to be in decline. Media focus tends to be on the colony collapse disorder phenomenon reported in honey bee colonies in the United States (Williams et al., 2010). However, wild pollinators, including bumblebees, also appear to have suffered dramatic declines in recent decades (Vanbergen and the Insect Pollinators Initiative, 2013). For many bees, particularly solitary species, very limited or no data is available (Brown and Paxton, 2009), making it difficult to accurately assess the scope of the decline. The evidence is slightly more comprehensive for bumblebees, particularly in the UK (Williams, 1982; Biesmeijer et al., 2006; Woodcock et al., 2016). The UK was historically home to 27 types of bumblebee; however, three of these are now extinct and a further seven have been given ‘Biodiversity Action Plan’ status due to dramatic declines across much of their historical range (UK BAP (Biodiversity Action Plan) priority terrestrial invertebrate species, 2007). The causes of the declines are complicated and driven by multiple interacting factors (Williams and Osborne, 2009; Potts et al., 2010; Goulson et al., 2015; Woodard, 2017). These include habitat loss and fragmentation resulting from agricultural intensification and landscape modification (Roulston and Goodell, 2011; Ollerton et al., 2014; Persson et al., 2015; Kamper et al., 2016), competition from commercial beekeeping and non-native species (Stout and Morales, 2009; Schweiger et al., 2010), parasites and diseases (Meeus et al., 2011; Fürst et al., 2014; McMahon et al., 2015; Grozinger and Flenniken, 2019), climate change (Kerr et al., 2015) and pesticide use (Woodcock et al., 2016; Gill and Raine, 2014; Stanley et al., 2015; Baron et al., 2017).

The relationship between pesticide use and bee decline is hotly debated (Connolly, 2013; Dicks, 2013; Walters, 2013; Cressey, 2017). Significantly more is known about the impact of pesticides on managed *A. mellifera* than wild bee species (Kiljanek et al., 2016). There have been reports of bumblebee decline following field pesticide application (Baron et al., 2017; Siviter et al., 2018), however, wild bee deaths from pesticide use are much more likely to go unnoticed (Goulson, 2010). On a European scale, a review of the risk of neonicotinoid insecticides on bee health by the European Food Safety Authority (EFSA) in 2012 (EFSA (European Food Safety Authority), 2012) led the European Commission to ban the use of three neonicotinoids (clothianidin, imidacloprid and thiamethoxam) on outdoor crops attractive to bees, and in cereals (except winter cereals). In December 2018, based on an updated risk assessment by EFSA, the ban was extended to all field crops. Banning these neonicotinoids has forced farmers to use alternative means of insect control on crops that previously received neonicotinoid seed treatments (Kathage et al., 2017). For example, growers of winter oil seed rape in the UK have switched to spray applications of pyrethroid insecticides to control cabbage stem flea beetle, despite there being considerable resistance issues (Hojland et al., 2015; Scott and Bilsborrow, 2018).

Presently, pesticide registration in the EU only requires contact and oral toxicity testing on honey bees (EFSA (European Food Safety Authority), 2013), but this will soon be extended to additional bee species, incl. *B. terrestris,* as it is increasingly recognized that current measures to mitigate honey bee exposure to insecticides may not be sufficient to protect wild bee species (Stoner, 2016). The European Crop Protection Association (ECPA) recently published a draft pesticide risk assessment document highlighting the lack of consideration of pesticide exposure for bumblebees and solitary bees (ECPA (European Crop Protection Association), 2017), whilst also acknowledging that test methods for these species are still only in the developmental stages. Subsequently, the Organisation for Economic Co-operation and Development (OECD) has released bumblebee protocols for acute contact (OECD (Organization for Economic Cooperation and Development), 2017a) and oral (OECD (Organization for Economic Cooperation and Development), 2017b) toxicity that follows the risk assessment scaffold for honey bees. In view of the differing impact of pesticides on different pollinator species, there is clearly now a need for better harmonization of test protocols.

**Fig. 1:**
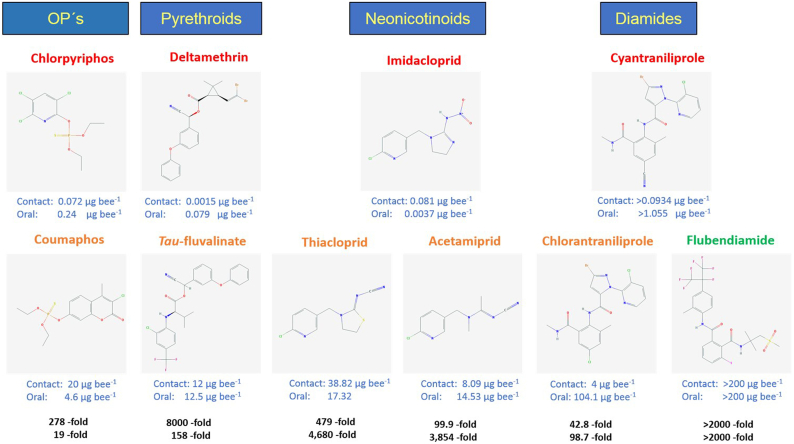
Acute 48 h contact and oral LD_50_ toxicity data for honey bee. Differential selectivity is found within most chemical classes of insecticides. Sources: ECOTOX (ECOTOX, database of the U.S. Environment Protection Agency, 2020) and AGRITOX (Agri-Tox, Database of the Agence Nationale de Se'curite´ Sanitaire de l'Alimentation, 2020) databases (data compiled by Sanchez-Bayo & Goka (Sanchez-Bayo and Goka, 2014)) and the Pesticide Properties Database (Lewis et al., 2016) (accessed at https://sitem.herts.ac.uk/aeru/ppdb/en/). Chemical structures were obtained from PubChem (Kim et al., 2016) (accessed at https://pubchem.ncbi.nlm.nih.gov/).

Whilst there are many ways of measuring acute toxicity/impact of pesticides on bees, the median lethal dose or LD_50_ is most commonly applied as the values are more directly comparable than field data (van der Steen, 2001). Furthermore, whilst acute LD_50_ values don't necessarily fully reflect toxicity in the field, they are a good way of standardizing and comparing between different test compounds. Whilst it has been noted that pesticides toxic to *A. mellifera* have broadly similar effects on bumblebees, it is not always appropriate to extrapolate and derive specific LD_50_ values for bumblebees (or any other bee species) from honey bee LD_50_ data (van der Steen et al., 2009; Heard et al., 2017), since some pesticides are clearly more toxic to honey bees, while others are more toxic to bumblebees (Arena and Sgolastra, 2014). Furthermore, for honey bees, it has been shown that differential sensitivity can also be found within a particular chemical class of insecticide (Johnson, 2015), including for the neonicotinoids, pyrethroids, organophosphates and diamides – see Fig. 1 for examples. In this study we find that intrinsic tolerance to certain insecticides within a chemical class extends to the bumblebee, *B. terrestris*, since this pollinator shows not only profound differences in its sensitivity to different neonicotinoids (thiacloprid, imidacloprid (as previously reported) (Manjon et al., 2018) and acetamiprid), but also, as in honey bees, to different compounds belonging to other insecticide classes, including the pyrethroids (deltamethrin, *tau*-fluvalinate) and the organophosphates (chlorpyrifos, coumaphos).

## 2 Materials and methods

### 2.1 Chemicals

Technical grade imidacloprid, thiacloprid and acetamiprid (neonicotinoids); *tau*-fluvalinate and deltamethrin (pyrethroids); chlorpyrifos-methyl and coumaphos (organophosphates); piperonyl butoxide (PBO) and triflumizole (synergists) were purchased from Sigma-Aldrich Company Ltd. (United Kingdom).

### 2.2 Bees

*Bombus terrestris audax* standard hives were obtained from Agralan Ltd. (Swindon, UK). Hives were maintained in constant darkness at 25 °C, 50% RH. The colonies were fed *ad libitum* on the nectar substitute, Biogluc® and pollen was supplied every 2 days to support larval development. For collecting bees for bioassay, entire hives were anesthetized using CO_2_ for the minimum time required to safely select and remove individual female workers. If required, bees were individually anesthetized again, prior to topical application of the synergist/insecticide, for the minimum amount of time required (5–10 s) to render them docile enough for safe handling. As the weight of individual *B. terrestris* workers varied, we limited our selection to individuals weighing between 150 and 250 mg. We also only selected healthy looking bees, *i.e.* those with intact legs and wings, and without bald patches and avoided using newly emerged callow bees in bioassays.

### 2.3 Insecticide bioassays

Acute contact and oral toxicity bioassays were based on the OECD Honey Bee Acute Contact Toxicity and Acute Oral Toxicity Test guidelines (OECD (Organisation for Economic Co-operation and Development), 1998a; OECD (Organisation for Economic Co-operation and Development), 1998b), with modifications as described. The bees were housed individually in disposable plastic tubes stoppered with cotton wool for the duration of the test period. For each set of bioassays, workers were selected from at least two colonies to limit potential colony effects. At least 5 concentrations of each technical grade insecticide were tested. The appropriate range of doses for each compound was determined using range-finder bioassays. In this way the lowest and highest doses of each insecticide needed to cause 0% and 100% mortality respectively where determined. The intermediate doses then followed a geometric progression, with the ratio between successive doses generally not exceeding 2. For each of the optimized concentrations at least 3 replicates, with ~10 bees per replicate were done.

For acute contact bioassays, test doses of insecticides were delivered in 2 μl acetone to the dorsal thorax of each anesthetized worker bee using a Burkhard Hand Micro Applicator (Burkhard Scientific Ltd., UK). Control bees were treated with just 2 μl 100% acetone. Concentrations of 10, 50, 100, 500 and 1000 ppm (equivalent to 0.02 to 2 μg/bee) were applied for imidacloprid; 3125, 6250, 12,500, 25,000 and 50,000 ppm (equivalent to 6.25 to 100 μg/bee) for acetamiprid, thiacloprid and coumaphos; 50, 100, 500, 1000 and 5000 ppm (equivalent to 0.1 to 10 μg/bee) for deltamethrin; 1250, 2500, 5000, 10,000, 20,000 ppm (equivalent to 2.5 to 40 μg/bee) for tau-fluvalinate; 50,100, 200, 400, 800 ppm (equivalent to 0.1 to 1.6 μg/bee) chlorpyriphos. Treated worker bees were fed *ad libitum* with a 50% (*w*/*v*) sucrose solution from disposable plastic syringes and kept in an incubator (25 °C, 55% RH, permanent darkness) for the duration of the test period.

Acute oral bioassays were only performed for the neonicotinoid insecticides, as these were the only compounds with sufficiently high solubility to enable LD_50_ values to be accurately determined. The three neonicotinoid compounds were dissolved in acetone up to the highest concentration possible, before being diluted to the appropriate test concentrations with 50% sucrose (*w*/*v*), to limit the volume of acetone consumed by the bees. Concentrations of 0.01, 0.1, 1, 10 and 100 ppm (equivalent to 0.0002 to 2 μg/bee) were fed for imidacloprid and 50, 100, 500, 1000 and 5000 ppm (equivalent to 0.2 to 100 μg/bee) for acetamiprid and thiacloprid. Worker bees were starved of sucrose solution for 2 h prior to testing to encourage feeding during the bioassay. Individual bees were supplied with 20 μl of insecticide/sucrose solution in a disposable plastic syringe. Control bees were supplied with a sucrose solution containing a volume of acetone matching that of the treatment containing the highest volume of acetone. After 4–6 h, the feeding syringes were assessed to ensure that only bees that had consumed the entire dosage were recorded in the bioassay. The selected bees were then fed 50% sucrose (*w*/*v*) *ad libitum*.

### 2.4 Synergist bioassays

The upper limit of each synergist that could be applied, without causing any mortality, was determined by initial range-finder tests. The final sub-lethal amount of synergist applied in bioassays was 20 μg/bee for PBO and triflumizole. In both contact and oral bioassays, the synergist was delivered in 2 μl acetone to the dorsal thorax of each anesthetized bee 1 h before topical application or feeding of insecticide (Iwasa et al., 2004) at the appropriate dosage as above, with the following exceptions: 1, 5, 10, 50, 100, 500 ppm (equivalent to 0.002 to 1 μg/bee) for deltamethrin; 5, 10, 50, 100, 500 ppm (equivalent to 0.01 to 1 μg/bee) for tau-fluvalinate. In addition to the standard acetone control, each bioassay included a synergist control treated with the maximum sublethal dose of each synergist.

### 2.5 Assessment and analysis

Mortality for both contact and oral toxicity bioassays was assessed 48 and 72 h after application of the insecticide. Bees were only assessed as ‘dead’ if they were truly dead, rather than moribund or severely affected, to eliminate subjectivity in the results. For each group of insecticide bioassays, a chi square test was used to verify that the control mortalities (which were consistently <10%) for each type of bioassay were not significantly different (see Supplementary data). The LD_50_ values (±95% confidence intervals) and slope were estimated for each insecticide using probit analysis (Finney, 1971), correcting the model for control mortality using generalized least squares (VSN International, 2015). Based on the contact LD_50_ value, the pesticide is classified as ‘practically non-toxic’ (LD_50_ > 100 μg/bee), slightly toxic (100 > LD_50_ > 11 μg/bee), ‘moderately toxic’ (11 > LD_50_ > 2/μg bee) or ‘highly toxic’ (LD_50_s <2 μg/bee) (Felton et al., 1986; USEPA (United States Environmental Protection Agency), 2014).

## 3 Results

Specific LD_50_ values, 95% confidence intervals, and synergism ratios for each compound tested, at 48 h and 72 h post treatment, are presented in Tables 1–3.

**Table 1:** Neonicotinoid acute contact and acute oral LD_50_ values (±95% confidence intervals), slope (±SE) and synergism ratio for *B. terrestris,* 48 and 72 h after application of insecticide. Synergism ratio is also shown, where synergists were used (piperonyl butoxide (PBO)). Compounds with LD_50_ values >100 μg/bee can be regarded as practically non-toxic (Felton et al., 1986); in such cases we could not achieve 100% mortality and could not generate LD_50_ values. When found to be non-toxic a limit test was carried out. Abbreviations used for toxicity classification: practically non-toxic (PNT), slightly toxic (ST), moderately toxic (MT), highly toxic (HT).

Application	Pesticide	Synergist	48 h					72 h					
			LD_50_ (μg/bee)	95% CI	Slope	± SE	Synergism Ratio	LD_50_ (μg/bee)	95% CI	Slope	± SE	Synergism Ratio	Toxicity
Topical	Acetamiprid	None	>100	n/a	n/a	n/a	n/a	>100	n/a	n/a	n/a	n/a	PNT
		PBO	>100	n/a	n/a	n/a	n/a	>100	n/a	n/a	n/a	n/a	PNT
	Imidacloprid	None	0.38	0.12–1.45	0.6	0.11	n/a	0.31	0.138–1.0	1.0	0.26	n/a	HT
		PBO	0.11	0.06–0.19	1.0	0.15	3.5	0.018	0.04–0.09	1.2	0.13	7.0	HT
	Thiacloprid	None	>100	n/a	n/a	n/a	n/a	>100	n/a	n/a	n/a	n/a	PNT
		PBO	>100	n/a	n/a	n/a	n/a	>100	n/a	n/a	n/a	n/a	PNT
Oral	Acetamiprid	None	13.13	9.27–18.63	2.9	0.59	n/a	12.88	9.18–18.03	2.85	0.56	n/a	ST
		PBO	9.03	5.44–13.07	2.4	0.51	1.5	8.45	5.47–11.69	2.6	0.52	1.6	MT
	Imidacloprid	None	0.038	0.012–0.075	1.5	0.44	n/a	0.042	0.015–0.079	1.8	0.54	n/a	HT
		PBO	0.032	0.016–0.05	1.9	0.41	1.2	0.023	0.0058–0.048	1.5	0.41	1.8	HT
	Thiacloprid	None	19.68	13.45–26.88	1.8	0.26	n/a	16.40	5.26–33.1	1.48	0.41	n/a	ST
		PBO	4.73	2.55–7.71	1.4	0.24	4.16	4.32	0.963–10.40	1.26	0.36	3.8	MT

### 3.1 Neonicotinoids

Topical application of imidacloprid was highly toxic to *B. terrestris* workers, and a low synergistic effect was seen when the bees were pre-treated with PBO (Table 1). In contrast, topical applications of acetamiprid or thiacloprid were practically non-toxic*.* For these two compounds, we tested doses of up to 100 μg/bee (limit test), with and without PBO, and were unable to obtain sufficient mortality to generate LD_50_ values. Overall there was a significant (> 250-fold) difference in toxicity between imidacloprid and acetamiprid or thiacloprid.

Oral application of the three neonicotinoids significantly increased their toxicity. Imidacloprid was again highly toxic, though this time less synergism was observed with PBO. Thiacloprid or acetamiprid alone were only slightly toxic but became moderately toxic with the addition of PBO. Again, there was a significant (> 250-fold) toxicity difference between imidacloprid and acetamiprid or thiacloprid (Table 1). The LD_50_ values for oral toxicities of all three neonicotinoids to bumblebees were virtually identical to those reported for honey bees (Fig. 1). LD_50_ values obtained for imidacloprid and acetamiprid were also very similar to those previously reported for *B. terrestris* (Sanchez-Bayo and Goka, 2014).

### 3.2 Pyrethroids

Topically applied deltamethrin was highly toxic to *B. terrestris,* whilst *tau-*fluvalinate was only slightly toxic (an approximate 17.5-fold difference). Only relatively mild synergism occurred for both compounds with the pre-application of triflumizole, but substantial synergism occurred with PBO, with synergism ratios of ~180 for deltamethrin and ~148 for *tau*-fluvalinate at 48 h post- insecticide application (Table 2). The topical LD_50_ of *tau*-fluvalinate to bumblebees is virtually identical to that reported for honey bees (Fig. 1). The recorded LD_50_ values for deltamethrin are also in line with those previously reported for *B. terrestris* (Sanchez-Bayo and Goka, 2014).

**Table 2:** Pyrethroid acute contact LD_50_ values (±95% confidence intervals), slope (±SE) and synergism ratio for *B. terrestris,* 48 and 72 h after application of insecticide. Synergism ratio is also shown, where synergists were used (piperonyl butoxide and triflumizole). Abbreviations used for toxicity classification: practically non-toxic (PNT), slightly toxic (ST), moderately toxic (MT), highly toxic (HT).

Application	Insecticide	Synergist	48 h					72 h					
			LD_50_ (μg/bee)	95% CI	Slope	± SE	Synergism Ratio	LD_50_ (μg/bee)	95% CI	Slope	± SE	Synergism Ratio	Toxicity
Topical	Deltamethrin	None	1.07	0.53-1.86	1.2	0.20	n/a	0.79	0.36-1.41	1.44	0.27	n/a	HT
		PBO	0.0060	0.0038-0.0084	2.6	0.54	179.6	0.0057	0.0036-0.0082	2.45	0.51	139.0	HT
		Triflumizole	0.19	0.1-0.34	1.3	0.22	5.6	0.15	0.059-0.288	1.75	0.43	5.2	HT
	Tau-fluvalinate	None	18.71	13.61-25.45	1.9	0.30	n/a	14.00	10.25-18.35	2.28	0.35	n/a	ST
		PBO	0.13	0.077-0.217	1.0	0.15	148.3	0.12	0.072-0.203	1.00	0.15	117.9	HT
		Triflumizole	12.75	8.16-19.3	1.5	0.28	1.5	5.72	3.18-8.31	1.75	0.33	2.4	MT/ST

### 3.3 Organophosphates

Topically applied coumaphos was practically non-toxic to *B. terrestris*, even after pre-application of PBO or triflumizole, with doses of up to 100 μg/bee giving insufficient mortality to generate LD_50_ values (Table 3). Chlorpyrifos-methyl in contrast was 150-fold more toxic to *B. terrestris* and classified as highly toxic. The previously reported LD_50_ value for chlorpyrifos-methyl (Sanchez-Bayo and Goka, 2014) was slightly lower than that recorded here, implying an even greater level of toxicity to *B. terrestris*.

**Table 3:** Organophosphate acute contact LD_50_ values (±95% confidence intervals), slope (±SE) and synergism ratio for *B. terrestris* 48 and 72 h after application of insecticide. Synergism ratio is also shown where synergists were used (piperonyl butoxide and triflumizole). Compounds with LD_50_ values >100 μg/bee can be regarded as practically non-toxic (Felton et al., 1986); in such cases we could not achieve 100% mortality and could not generate LD_50_ values. When found to be non-toxic a limit test was performed. Abbreviations used for toxicity classification: practically non-toxic (PNT), slightly toxic (ST), moderately toxic (MT), highly toxic (HT).

Application	Pesticide	Synergist	48 h					72 h					
			LD_50_ (μg/bee)	95% CI	Slope	± SE	Synergism Ratio	LD_50_ (μg/bee)	95% CI	Slope	± SE	Synergism Ratio	Toxicity
Topical	Chlorpyrifos	None	0.64	0.5-0.78	4.5	0.94	n/a	0.57	0.34-0.82	3.6	1.01	n/a	HT
		PBO	0.44	0.31-0.66	2.3	0.50	1.4	0.39	0.28-0.54	2.9	0.64	1.4	HT
	Coumaphos	None	>100	n/a	n/a	n/a	n/a	>100	n/a	n/a	n/a	n/a	PNT
		PBO	>100	n/a	n/a	n/a	n/a	>100	n/a	n/a	n/a	n/a	PNT
		Triflumizole	>100	n/a	n/a	n/a	n/a	>100	n/a	n/a	n/a	n/a	PNT

**Fig. 2:**
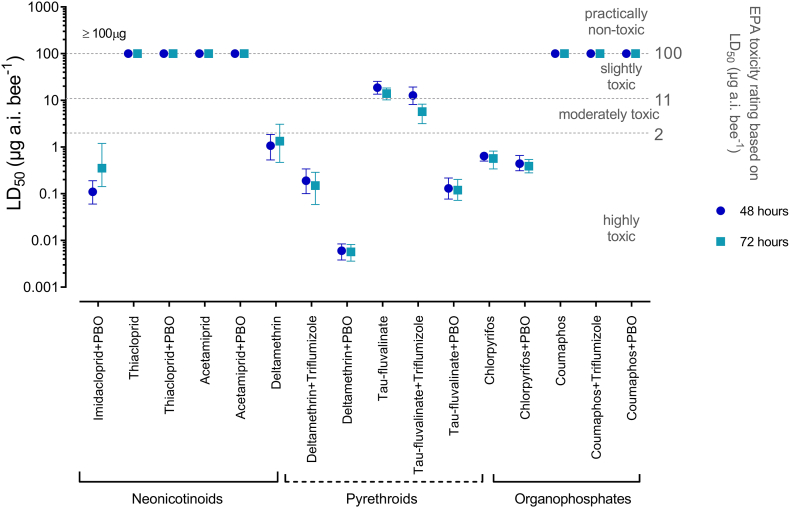
LD_50_ values for topical insecticide bioassays. Thresholds are depicted according to EPA toxicity ratings (USEPA (USEPA (United States Environmental Protection Agency), 2014)). Error bars display 95% CLs (*n* = 4).

**Fig. 3:**
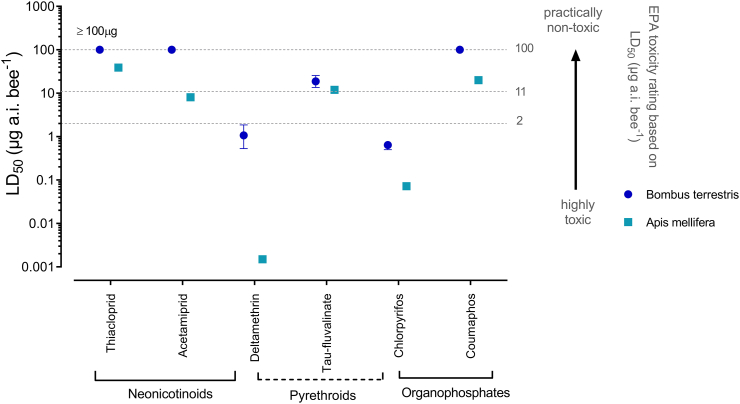
Comparison of bumblebee and honeybee LD_50_ values obtained by topical bioassays. Thresholds are depicted according to EPA toxicity ratings (USEPA (USEPA (United States Environmental Protection Agency), 2014)). Error bars display 95% CLs (n = 4).

The data reported for the bumblebee, *B. terrestris,* and the representative insecticides in the three chemical classes neonicotinoids, pyrethroids and organophosphates are further summarized in Fig. 2 and compared with previously recorded data for honey bees in Fig. 3.

## 4 Discussion

Neonicotinoids currently make up 30% of insecticide sales worldwide (Simon-Delso et al., 2015). There are at least seven compounds commercially available, including the *N*-nitroguanidines imidacloprid, thiamethoxam (metabolized to clothianidin in the plant, insect and environment) (Nauen et al., 2003), clothianidin and dinotefuran, the nitromethylene nitenpyram and the *N*-cyanoamidines acetamiprid and thiacloprid (Jeschke et al., 2011). Initially, five of these, clothianidin, imidacloprid, thiamethoxam, thiacloprid and acetamiprid, were approved for use in the European Union, but several of these have now been banned due to their perceived detrimental effects on honey bees. Concern over effects on plant pollinators has mostly focused on the *N*-nitroguanidines because they are most commonly used, they have greater honey bee toxicity (Manjon et al., 2018; Iwasa et al., 2004; Nauen et al., 2001) and are often applied as seed treatments so can be present in the pollen and nectar of treated crops (Rundlöf et al., 2015). The *N*-cyanoamidines are generally considered to be safer for honey bees (*e.g.* see Schmuck, 2001) (Schmuck, 2001), with oral and contact toxicities 2–3 orders of magnitude lower than for the *N*-nitroguanidines. In a recent study (Manjon et al., 2018), Manjon et al. demonstrated that the nicotinic acetylcholine receptors of honey bees do not distinguish between thiacloprid and imidacloprid, and that the target is equally sensitive to both compounds. They subsequently identified a single honey bee cytochrome P450 enzyme, CYP9Q3 belonging to the CYP9Q family, capable of efficiently detoxifying thiacloprid and acetamiprid to less toxic metabolites before they reach the receptor. Two close paralogs of CYP9Q3 in *B. terrestris,* CYP9Q4 and CYP9Q6, have also been identified as a metabolizer of thiacloprid and acetamiprid (Manjon et al., 2018; Troczka et al., 2019). In contrast, these CYP9Q P450s were poor imidacloprid metabolizers. The results reported here demonstrate that as for honey bees, both thiacloprid and acetamiprid are not acutely toxic to bumblebees, as determined by median lethal dose (LD_50_). Furthermore, application of PBO had a minimal effect on LD_50_ values, suggesting that although considered a generalist P450 inhibitor it is not an effective inhibitor of CYP9Q4 or CYP9Q6. What is clear from the present study, and that of Manjon et al. (Manjon et al., 2018) is that the neonicotinoids thiacloprid and acetamiprid are much less toxic to both honeybees and buff-tailed bumblebees than imidacloprid, even though they all target nicotinic acetylcholine receptors. These results further suggest that the new insect nicotinic acetylcholine receptor modulators which have been developed over the past ten years, which include the sulfoximines (Zhu et al., 2011) exemplified by the systemic insecticide sulfoxaflor (Sparks et al., 2013), the butenolides such as flupyradifurone (Nauen et al., 2015) and the mesoionics exemplified by triflumezopyrim (Cordova et al., 2016), and flupyrimin (Onozaki et al., 2017) should each be assessed for their toxicity to bees as individual compounds and not assumed to have the same toxicity as the *N*-nitroguanidine neonicotinoids. Recently, it was shown that sulfoxaflor may potentially harm bumblebees (Siviter et al., 2018), however, no data is currently available for other sulfoximine insecticides. Equivalent studies have not to date been conducted on the other competitive modulators of nicotinic acetylcholine receptors listed above, which initial toxicology reports suggest are relatively bee safe.

Since the recent phased ban of several neonicotinoid compounds across Europe, pyrethroid insecticides are now being more intensively used. Pyrethroids have for many decades been widely applied in agriculture, and in urban areas for vector control. They are highly lipophilic, broad-spectrum insecticides, and exert their insecticidal effect by prolonging the open phase of the voltage-gated sodium channel in neurons (Naumann, 1990). Currently, the impact of pyrethroids on bumblebee pollinators is poorly characterized. Chronic exposure to λ-cyhalothrin has been reported to have a significant impact on worker size, a key aspect of bumblebee colony function, particularly under conditions of limited food resources (Baron et al., 2014), and bumblebee mortality under field conditions (Ceuppens et al., 2015). In contrast the pyrethroid *tau*-fluvalinate is considered to be bee safe even when sprayed in the full-flight phase of bumblebees (Sterk and Kolokytha, 2015). However, repeated application for control of in-hive Varroa mites can contaminate wax to levels as high as 200 ppm, leading to issues with larval survival (Berry et al., 2013; Zhu et al., 2014). Our study confirms that, as shown for other *alpha*-cyano substituted pyrethroids, deltamethrin is highly toxic to *B. terrestris,* while *tau-*fluvalinate, which is routinely employed in apiaries to control Varroa, is only slightly toxic (an approximate 17.5-fold difference). Both pyrethroids are synergized by the application of PBO. A previous study (Mao et al., 2011) has indicated that in honey bees the P450s CYP9Q1, CYP9Q2, and CYP9Q3 are capable of metabolizing *tau*-fluvalinate, likely explaining the low toxicity of this compound to this species. The potent synergism seen between *tau*-fluvalinate and PBO suggests that, as previously reported for honeybees, P450s are especially important in the detoxification of this pyrethroid in *B. terrestris*.

The organophosphate chlorpyrifos, which is used to treat a number of crops on which bumblebees forage, including grasslands, cranberries, topfruit, oilseed rape, and potatoes, is confirmed in our study as being highly toxic to bumblebees. Organophosphorus insecticides in general function by inhibiting the enzyme acetylcholinesterase, which mediates the transmission of nerve signals (Fukuto, 1990). In contrast to chlorpyrifos, coumaphos, which is often used in hives to control Varroa, is relatively bee safe. With repeated use, coumaphos, considered to have low acute toxicity, can build up in the wax of honey bee colonies to concentrations as high as 90 ppm, again leading to issues with larval mortality of both queens and workers (Berry et al., 2013; Zhu et al., 2014). In common with *tau*-fluvalinate, the honey bee CYP9Q1–3 P450s have been shown to be capable of metabolizing coumaphos (Mao et al., 2011). These three P450s therefore seem to play a key role in the honey bees' defense against insecticides, being capable of metabolizing compounds belonging to at least three different insecticide classes, as well as natural diet-derived phytochemicals such as quercetin. The CYP9Q family in general, may therefore be critically important in defining the sensitivity of eusocial bees, including *B. terrestris,* to xenobiotic insult (Manjon et al., 2018). Thus, there is a growing body of evidence that metabolism by different P450s in different bee species influences the toxicity of insecticides and that extrapolations from one combination of chemical/bee species to another combination should not be made.

## 5 Conclusions

The data reported here show that in all three of the chemical classes tested there are significant differences in toxicity towards *B. terrestris*. For the neonicotinoids, imidacloprid is highly toxic but thiacloprid and acetamiprid are practically non-toxic, for pyrethroids deltamethrin is highly toxic but tau-fluvalinate is only slightly toxic and for the organophosphates chlorpyrifos in highly toxic but coumaphos is practically non-toxic. These results are similar to those reported for honey bees and are important because they demonstrate that a chemical class of insecticide does not indicate its potential toxicity and that decisions on regulation of use should be based on the properties of each compound, rather than its chemical class or its target site within the insect. Our results do not however address ‘sublethal’ effects that may result in eventual death, nor can they be extrapolated to make predictions for less resilient bee species (*e.g.* see Arena & Scolastro) (Arena and Sgolastra, 2014).

## Declaration of Competing Interests

This study received funding from Bayer AG, a manufacturer of neonicotinoid insecticides.

## Appendix A Supplementary data

Supplementary material Image 1

## References

[bb0005] Agri-Tox, Database of the Agence Nationale de Se’´curite´ Sanitaire de l’’Alimentation (2020). de l’Environnement et du Travail in France.

[bb0010] Arena M., Sgolastra F. (2014). A meta-analysis comparing the sensitivity of bees to pesticides. Ecotoxicology.

[bb0015] Baron G.L., Raine N.E., Brown M.J.F. (2014). Impact of chronic exposure to a pyrethroid pesticide on bumblebees and interactions with a trypanosome parasite. J. Applied Ecology.

[bb0020] Baron G.L., Jansen V.A., Brown M.J., Raine N.E. (2017). Pesticide reduces bumblebee colony initiation and increases probability of population extinction. Nat. Ecol. Evol..

[bb0025] Berry J.A., Hood W.M., Pietravalle S., Delaplane K.S. (2013). Field-level sublethal effects of approved bee hive chemicals on honey bees (*Apis mellifera* L). PLoS One.

[bb0030] Biesmeijer J.C., Roberts S.P., Reemer M., Ohlemülle R., Edwards M., Peeters T., Shaffers A.P., Potts S.G., Kleukers R., Thomas C.D., Settele J., Kunin W.E. (2006). Parallel declines in pollinators and insect-pollinated plants in Britain and the Netherlands. Science.

[bb0035] Brown M.J.F., Paxton R.J. (2009). The conservation of bees: a global perspective. Apidologie.

[bb0040] Ceuppens B., Eeraerts M., Vleugels T., Cnops G., Roldan-Ruiz I., Smagghe G. (2015). Effects of dietary lambda-cyhalothrin exposure on bumblebee survival, reproduction, and foraging behaviour in laboratory and greenhouse. J. Pest. Sci..

[bb0045] Connolly C. (2013). The risk of insecticides to pollinating insects. Commun. Integr. Biol..

[bb0050] Corbet S.A., Fussell M., Ake R., Fraser A., Gunson C., Savage A., Smith K. (1993). Temperature and the pollinating activity of social bees. Ecol. Entomol..

[bb0055] Cordova D., Benner E.A., Schroeder M.E., Holyoke C.W., Zhang W., Pahutski T.F., Leighty R.M., Vincent D.R., Hamm J.C. (2016). Mode of action of triflumezopyrim: a novel mesoionic insecticide which inhibits the nicotinic acetylcholine receptor. Insect Biochem. Mol. Biol..

[bb0060] Cressey D. (2017). The bitter battle over the world’s most popular insecticides. Nature.

[bb0065] Delaplane K.S., Mayer D.F. (2000). Crop Pollination by Bees.

[bb0070] Dicks L. (2013). Bees, lies and evidence-based policy. Nature.

[bb0075] ECOTOX, database of the U.S. Environment Protection Agency, Washington (DC). http://cfpub.epa.gov/ecotox/ (accessed 26/02/2020), 2020.

[bb0080] ECPA (European Crop Protection Association) (2017). Proposal for a protective and workable regulatory European bee risk assessment scheme based on the EFSA bee guidance and other new data and available approaches.

[bb0085] EFSA (European Food Safety Authority) (2012). Statement on the findings in recent studies investigating sub-lethal effects in bees of some neonicotinoids in consideration of the uses currently authorised in Europe. EFSA J..

[bb0090] EFSA (European Food Safety Authority) (2013). EFSA guidance document on the risk assessment of plant protection products on bees (*Apis mellifera*, *Bombus* spp. and solitary bees). EFSA Journal 11.

[bb0095] Felton J.C., Oomen P.A., Stevenson J.H. (1986). Toxicity and hazard of pesticides to honeybees: harmonization of test methods. Bee World.

[bb0100] Finney D.J. (1971). Probit Analysis.

[bb0105] Fukuto T.R. (1990). Mechanism of action of organophosphorus and carbamate insecticides. Environ. Health Perspect..

[bb0110] Fürst M.A., McMahon D.P., Osborne J.L., Paxton R.J., Brown M.J.F. (2014). Disease associations between honeybees and bumblebees as a threat to wild pollinators. Nature.

[bb0115] Gallai N., Salles J.-M., Settele J., Vaissiere B.E. (2009). Economic valuation of the vulnerability of world agriculture confronted with pollinator decline. Ecol. Econ..

[bb0120] Garibaldi L.A., Steffan-Dewenter I., Winfree R., Aizen M.A., Bommarco R., Cunningham S.A., Klein A.M. (2013). Wild pollinators enhance fruit set of crops regardless of honey bee abundance. Science.

[bb0125] Gill R.J., Raine N.E. (2014). Chronic impairment of bumblebee natural foraging behaviour induced by sublethal pesticide exposure. Funct. Ecol..

[bb0130] Goulson D. (2010). Bumblebees: Behaviour, Ecology, and Conservation (Second Ed).

[bb0135] Goulson D., Nicholls E., Botıas C., Rotheray E.L. (2015). Bee declines driven by combined stress from parasites, pesticides, and lack of flowers. Science.

[bb0140] Grozinger C.M., Flenniken M. (2019). Bee viruses: ecology, pathogenicity, and impacts. Annu. Rev. Entomol..

[bb0145] Heard M.S., Baas J., Dorne J.-L., Lahive E., Robinson A.G., Rortais A., Spurgeon D.J., Svendsen C., Hesketh H. (2017). Comparative toxicity of pesticides and environmental contaminants in bees: are honey bees a useful proxy for wild bee species?. Sci. Total Environ..

[bb0150] Hojland D.H., Nauen R., Foster S.P., Williamson M.S., Kristensen M. (2015). Incidence, spread and mechanism of pyrethroid resistance in European populations of the cabbage stem flea beetle, *Psylliodes chrysocephala* L. PLoS One.

[bb0155] Iwasa T., Motoyama N., Ambrose J.T., Roe R.M. (2004). Mechanism for the differential toxicity of neonicotinoid insecticides in the honey bee, *Apis mellifera*. Crop Prot..

[bb0160] Jeschke P., Nauen R., Schindler M., Elbert A. (2011). Overview of the status and global strategy for neonicotinoids. J. Agric. Food Chem..

[bb0165] Johnson R.M. (2015). Honey bee toxicology. Annu. Rev. Entomol..

[bb0170] Kamper W., Werner P.K., Hilpert A., Westphal C., Blutghen N., Eltz T., Leonhardt S.D. (2016). How landscape, pollen intake and pollen quality affect colony growth in *Bombus terrestris*. Landsc. Ecol..

[bb0175] Kathage J., Castanera P., Alonso-Prados J.L., Gomez-Barbero M., Rodriguez-Cerezo E. (2017). The impact of restrictions on neonicotinoid and fipronil insecticides on pest management in maize, oilseed rape and sunflower in eight European Union regions. Pest Manag. Sci..

[bb0180] Kerr J.T., Pindar A., Galpern P., Packer L., Potts S.G., Roberts S.M., Rasmont P., Schweigher O., Colla S.R., Richardson L.L., Wagner D.L., Gall L.F., Sikesm D.S., Patoja A. (2015). Climate change impacts on bumblebees converge across continents. Science.

[bb0185] Kiljanek T., Niewiadowska A., Posyniak A. (2016). Pesticide poisoning of honeybees: a review of symptoms, incident classification, and causes of poisoning. J. Apic. Sci..

[bb0190] Kim S., Thiessen P.A., Bolton E.E., Chen J., Fu G., Gindulyte A., Han L., He J., He S., Shoemaker B.A., Wang J., Yu B., Zhang J., Bryant S.H. (2016). PubChem substance and compound databases. Nucleic Acids Res..

[bb0195] Kleijn D., Winfree R., Bartomeus I., Carvalheiro L.G., Henry M., Isaacs R., Potts S.G. (2015). Delivery of crop pollination services is an insufficient argument for wild pollinator conservation. Nature communications.

[bb0200] Lautenbach S., Seppelt R., Liebscher J., Dormann C.F. (2012). Spatial and temporal trends of global pollination benefit. PLoS One.

[bb0205] Lewis K.A., Tzilivakis J., Warner D., Green A. (2016). An international database for pesticide risk assessments and management. Human and Ecological Risk Assessment: An International Journal.

[bb0210] Manjon C., Troczka B.J., Zaworra M., Beadle K., Randall E., Hertlein G., Singh K.S., Zimmer C.T., Homem R.A., Lueke B., Reid R., Kor L., Kohler M., Benting J., Williamson M.S., Davies T.G.E., Field L.M., Bass C., Nauen R. (2018). Unravelling the molecular determinants of bee sensitivity to neonicotinoid insecticides. Curr. Biol..

[bb0215] Mao W., Schuler M.A., Berenbaum M.R. (2011). CYP9Q-mediated detoxification of acaricides in the honey bee (*Apis mellifera*). Proc. Natl. Acad. Sci. U. S. A..

[bb0220] McMahon D.P., Fürst M.A., Caspar J., Theodorou P., Brown M.J.F., Paxton R.J. (2015). A sting in the spit: widespread cross-infection of multiple RNA viruses across wild and managed bees. J. Anim. Ecol..

[bb0225] Meeus I., Brown M.J.F., de Graaf D.C., Smagghe G. (2011). Effects of invasive parasites on bumble bee declines. Conserv. Biol..

[bb0230] Nauen R., Ebbinghaus-Kintscher U., Schmuck R. (2001). Toxicity and nicotinic acetylcholine receptor interaction of imidacloprid and its metabolites in *Apis mellifera* (Hymenoptera: Apidae). Pest Manag. Sci..

[bb0235] Nauen R., Ebbinghaus-Kintscher U., Salgado V.L., Kaussmann M. (2003). Thiamethoxam is a neonicotinoid precursor converted to clothianidin in insects and plants. Pestic. Biochem. Physiol..

[bb0240] Nauen R., Jeschke P., Velten R., Beck M.E., Ebbinghaus-Kintscher U., Thielert W., Wolfel K., Haas M., Kunz K., Raupach G. (2015). Flupyradifurone: a brief profile of a new butenolide insecticide. Pest Manag. Sci..

[bb0245] Naumann K. (1990). Synthetic Pyrethroid Insecticides: Structures and Properties.

[bb0250] OECD (Organisation for Economic Co-operation and Development) (1998). Test No. 213: Honeybees, Acute Oral Toxicity Test. http://0.0.0.26/02/2020.

[bb0255] OECD (Organisation for Economic Co-operation and Development) (1998). Test No. 214: Honeybees, Acute Contact Toxicity Test. http://0.0.0.26/02/2020.

[bb0260] OECD (Organization for Economic Cooperation and Development) (2017). OECD guidelines for the testing of chemicals. Bumblebee, acute contact toxicity test.

[bb0265] OECD (Organization for Economic Cooperation and Development) (2017). OECD guidelines for the testing of chemicals. Bumblebee, acute oral toxicity test.

[bb0270] Ollerton J., Erenler H., Edwards M., Crockett R. (2014). Extinctions of aculeate pollinators in Britain and the role of large-scale agricultural changes. Nature.

[bb0275] Onozaki Y., Horikoshi R., Ohno I., Kitsuda S., Durkin K.A., Suzuki T., Asahara C., Hiroki N., Komabashiri R., Shimizu R., Furutani S., Ihara M., Matsuda K., Mitomi M., Kagabu S., Uomoto K., Tomizawa M. (2017). Flupyrimin: a novel insecticide acting at the nicotinic acetylcholine receptors. J. Agric. Food Chem..

[bb0280] Persson A.S., Rundlöf M., Cloughm Y., Smith H.G. (2015). Bumble bees show trait-dependent vulnerability to landscape simplification. Biodivers. Conserv..

[bb0285] Plowright C.M.S., Plowright R.C. (1997). The advantage of short tongues in bumble bees (*Bombus*): analyses of species distributions according to flower corolla depth, and of working speeds on white clover. Can. Entomol..

[bb0290] Potts S.G., Biesmeijer J.C., Kremen C., Neumann P., Schweiger O., Kunin W.E. (2010). Global pollinator declines: trends, impacts and drivers. Trends Ecol. Evol..

[bb0295] Roulston T.H., Goodell K. (2011). The role of resources and risks in regulating wild bee populations. Annu. Rev. Entomol..

[bb0300] Rundlöf M., Andersson G.K., Bommarco R., Fries I., Hederstrom V., Herbertsson L., Jonsson O., Klatt B.K., Pedersen T.R., Yourstone J., Smith H.G. (2015). Seed coating with a neonicotinoid insecticide negatively affects wild bees. Nature.

[bb0305] Sanchez-Bayo F., Goka K. (2014). Pesticide residues and bees - a risk assessment. PLoS One.

[bb0310] Schmuck R. (2001). Ecotoxicological profile of the insecticide thiacloprid. Pflanzenschutz Nachrichten-Bayer-English Edition.

[bb0315] Schweiger O., Biesmeijer J.C., Bommarco R., Hickler T., Hulme P.E., Klotz S., Kühn I., Moora M., Nielsen A., Ohlemüller R., Petanidou T., Potts S.G., Pyšek P., Stout J.C., Sykes M.T., Tscheulin T., Vilà M., Walther G.R., Westphal C., Winter M., Zobel M., Settele J. (2010). Multiple stressors on biotic interactions: how climate change and alien species interact to affect pollination. Biol. Rev. Camb. Philos. Soc..

[bb0320] Scott C., Bilsborrow P.E. (2018). The impact of the EU neonicotinoid seed-dressing ban on oilseed rape production in England. Pest Manag. Sci..

[bb0325] Simon-Delso N., Amaral-Rogers V., Belzunces L.P., Bonmatin J.M., Chagnon M., Downs C., Furlan L., Gibbons D.W., Giorio C., Giroloami V., Goulson D., Kreutzweiser D.P., Krupke C.H., Liess M., Long E., McField M., Mineau P., Mitchell E.A.D., Morrissey C.A., Noome D.A., Pisa L., Settele J., Stark J.D., Tapparo A., Van Dyck H., Van Praagh J.P., Van der Sluijs J.P., Whitehorn P.R., Wiemers M. (2015). Systemic insecticides (neonicotinoids and fipronil): trends, uses, mode of action and metabolites. Environ. Sci. Pollut. Res..

[bb0330] Siviter H., Brown M.J.F., Leadbeater E. (2018). Sulfoxaflor exposure reduces bumblebee reproductive success. Nature.

[bb0335] Sparks T.C., Watson G.B., Loso M.R., Geng C., Babcock J.M., Thomas J.D. (2013). Sulfoxaflor and the sulfoximine insecticides: chemistry, mode of action and basis for efficacy on resistant insects. Pestic. Biochem. Physiol..

[bb0340] Stanghellini M.S., Ambrose J.T., Schultheis J.R. (2002). Diurnal activity, floral visitation and pollen deposition by honey bees and bumble bees on field-grown cucumber and watermelon. J. Apic. Res..

[bb0345] Stanley D.A., Garratt M.P.D., Wickens J.B., Wickens V.J., Potts S.G., Raine N.E. (2015). Neonicotinoid pesticide exposure impairs crop pollination services provided by bumblebees. Nature.

[bb0350] van der Steen J.J.M. (2001). Review of the methods to determine the hazard and toxicity of pesticides to bumblebees. Apidologie.

[bb0355] van der Steen J.J.M., Bortolotti L., Chauzat M. (2009). Can pesticide acute toxicity for bumblebees be derived from honeybee LD50 values?. Julius-Kühn-Archiv.

[bb0360] Sterk G.M., Kolokytha P.D. (2015). New insights of side-effects of tau-fluvalinate on biological agents and pollinators. Commun. Agric. Appl. Biol. Sci..

[bb0365] Stoner K.A. (2016). Current pesticide risk assessment protocols do not adequately address differences between honey bees (*Apis mellifera*) and bumble bees (Bombus spp.). Front. in Environ. Sci..

[bb0370] Stout J.C., Morales C.L. (2009). Ecological impacts of invasive alien species on bees. Apidologie.

[bb0375] Troczka B.J., Homem R.A., Reid R., Beadle K., Kohler M., Zaworra M., Field L.M., Williamson M.S., Nauen R., Bass C., Davies T.G.E. (2019). Identification and functional characterisation of a novel N-cyanoamidine neonicotinoid metabolising cytochrome P450, CYP9Q6, from the buff-tailed bumblebee *Bombus terrestris*. Insect Biochem. Mol. Biol..

[bb0380] UK BAP (Biodiversity Action Plan) priority terrestrial invertebrate species (2007). Joint Nature Conservation Committee.

[bb0385] USEPA (United States Environmental Protection Agency) (2014). Guidance for Assessing Pesticide Risks to Bees.

[bb0390] Vanbergen A.J., the Insect Pollinators Initiative (2013). Threats to an ecosystem service: pressures on pollinators. Front. Ecol. Environ..

[bb0395] Velthuis H.H.W., van Doorn A. (2006). A century of advances in bumblebee domestication and the economic and environmental aspects of its commercialization for pollination. Apidologie.

[bb0400] VSN International (2015). Genstat for Windows.

[bb0405] Walters K. (2013). Data, data everywhere but we don't know what to think? Neonicotinoid insecticides and pollinators. Outlook Pest Manag..

[bb0410] Williams G.R., Tarpy D.R., van Engelsdorp D., Chauzat M.P., Cox-Foster D.L., Delaplane K.S., Neumann P., Pettis J.S., Rogers R.E., Shutler D. (2010). Colony collapse disorder in context. Bioessays.

[bb0415] Williams P.H. (1982). The distribution and decline of British bumble bees (Bombus Latr.). J. Apic. Res..

[bb0420] Williams P.H., Osborne J.L. (2009). Bumblebee vulnerability and conservation world-wide. Apidologie.

[bb0425] Woodard S.H. (2017). Bumble bee ecophysiology: integrating the changing environment and the organism. Curr. Opin. Insect Sci..

[bb0430] Woodcock B.A., Isaac N.J.B., Bullock J.M., Roy D.B., Garthwaite D.G., Crowe A., Pywell R.F. (2016). Impacts of neonicotinoid use on long-term population changes in wild bees in England. Nat. Commun..

[bb0435] Zhu W., Schmehl D.R., Mullin C.A., Frazier J.L. (2014). Four common pesticides, their mixtures and a formulation solvent in the hive environment have high oral toxicity to honey bee larvae. PLoS One.

[bb0440] Zhu Y., Loso M.R., Watson G.B., Sparks T.C., Rogers R.B., Huang J.X., Gerwick B.C., Babcock J.M., Kelley D., Hegde V.B., Nugent B.M., Renga J.M., Denholm I., Gorman K., DeBoer G.J., Hasler J., Meade T., Thomas J.D. (2011). Discovery and characterization of sulfoxaflor, a novel insecticide targeting sap-feeding pests. J. Agric. Food Chem..

