# Supplementary figures and images for: Assessing the acute toxicity of insecticides to the buff-tailed bumblebee (Bombus terrestris audax)
Source: Pestic Biochem Physiol. 2020 Jun;166:104562. doi: 10.1016/j.pestbp.2020.104562 (PMC7294345; doi:10.1016/j.pestbp.2020.104562)

## Slide 1
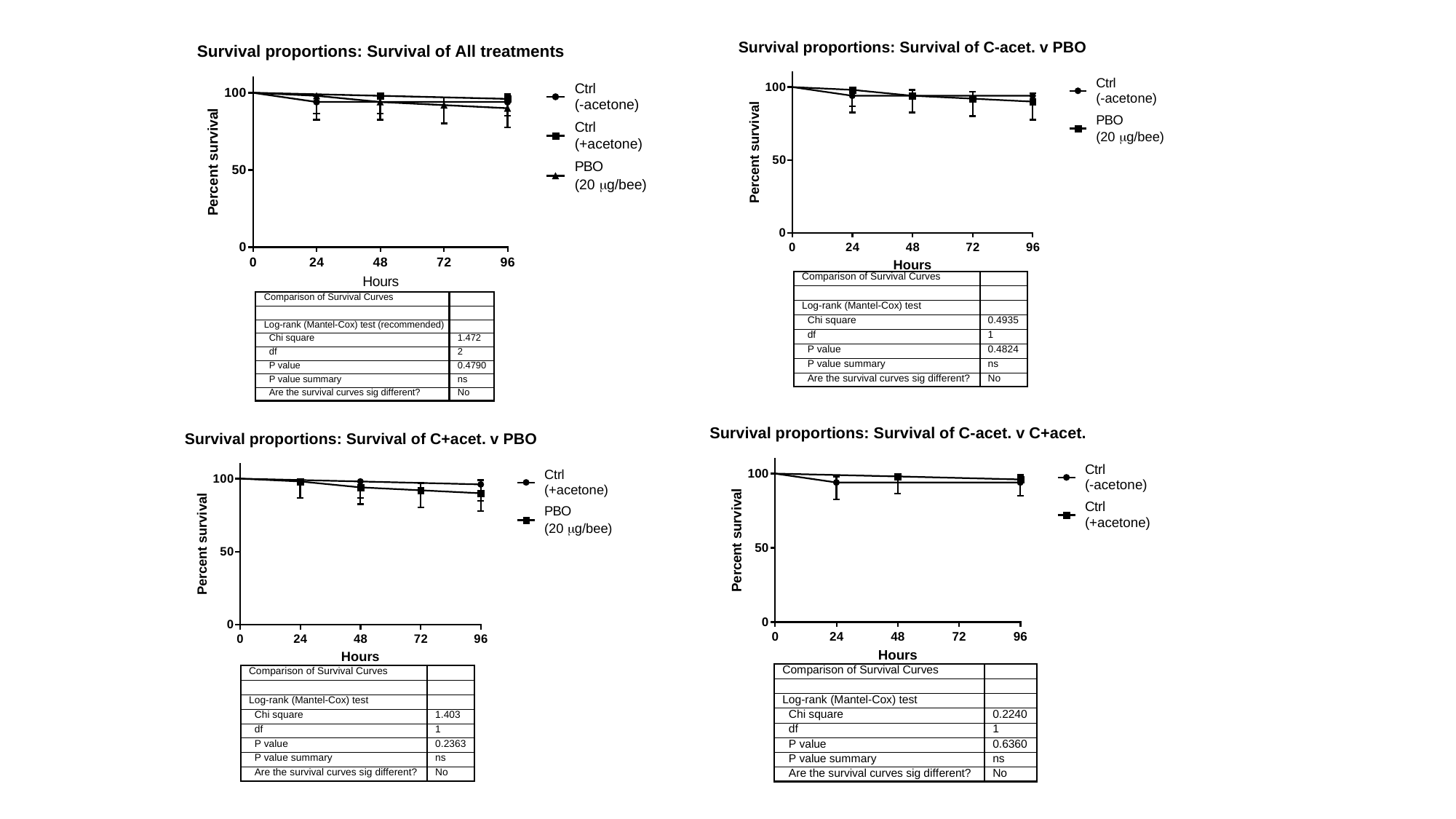

Supplement: Supplementary file 1 — Supplementary material [file mmc1.pptx]
